# Effects of Selective iNOS Inhibitor on Spatial Memory in Recovered and Non-Recovered Ketamine Induced Anesthesia

**Published:** 2011

**Authors:** Kaveh Tabrizian, Sheyda Najafi, Maryam Belaran, Ali Hosseini-Sharifabad, Kian Azami, Maliheh Soodi, Ali Kazemi, Abbas Kebriaeezadeh, Mohammad Sharifzadeh

**Affiliations:** a *Department of Pharmacology and Toxicology.*; b *Pharmaceutical Sciences Research Center, Faculty of Pharmacy, Tehran University of Medical Sciences, P.O. Box 14155-6451, Tehran, Iran*; c *Department of Toxicology, Faculty of Medical Sciences, Tarbiat Modares University, Tehran, Iran.*

**Keywords:** Inducible nitric oxide synthase, Anesthesia, CA1 region, Morris water maze, Spatial memory, Protein kinase

## Abstract

Nitric oxide (NO) is thought to be involved in spatial learning and memory in several brain areas such as hippocampus. This study examined the effects of post-training intrahippocampal microinjections of 1400W as a selective iNOS inhibitor on spatial memory, in anesthetized and non-anesthetized situations in rats. In the present work, 4-day training trials of animals were conducted. Spatial memory was tested 48 hours after the drug infusions. For microinjection of 1400W into CA1 region of the hippocampus in conscious animals, guide cannula was implanted into the CA1 area and 1400W was infused after recovery from surgical anesthesia. In anesthetized animals, 1400W was microinjected directly into CA1 region by Hamilton syringe during anesthesia. After completion of training, 1400W (10, 50 and 100 μM/side) were microinjected bilaterally (1 μL/side) and testing trials were performed 48 h after drug infusions in both groups of cannulated and non-cannulated rats. Significant reduction was observed in escape latency and traveled distance in animals that received 1400W (100 μM/side, *p < 0.05) via cannula after recovery in comparison with control group. Also, microinjection of 1400W (100 μM/side) in post recovery phase caused a significant (***p < 0.001) reduction in time and distance of finding the hidden platform in comparison with anesthetized situation. These findings suggest that 1400W has a significant improvement on spatial memory and memory enhancement induced by iNOS inhibitor can be affected by anesthesia in a period of time.

## Introduction

Memory is a complicated function with poorly understood findings. In processing of multiple phases of memory phenomena, various distributed neuronal systems, gene expression, protein synthesis and structural alterations of signaling pathways are involved (1, 2).

The critical role of the hippocampus in memory has been shown in many studies (3-7). After reporting of sever amnesia following temporal lobe resection of hippocampus, a growing body of researches has directed at evaluation of the functional role of the hippocampus in different kinds of memory including spatial memory, declarative memory, explicit memory and relational memory (3, 8).

Various studies demonstrate the effects of anesthetic agents on memory (2, 9-14). The impairment effects of general anesthetics on memory function have been indicated in the previous experiments (2, 14). However some reports show the memory facilitation of general anesthetics during consolidation phase (2, 15).

A substantial body of evidence has suggested that nitric oxide (NO) has an important role in synaptic plasticity in different brain areas such as cerebellum and hippocampus )16, 17(. But, findings about the importance of hippocampal nitric oxide in spatial learning and memory are controversial )16(*. *Nitric oxide synthase (NOS) exists in at least three isoforms including eNOS (endothelial NOS), nNOS (neuronal isozyme of NOS) and iNOS (inducible NOS). iNOS is mediated independently to calcium, but eNOS and nNOS are both stimulated in a calcium dependent manner (16). All nitric oxide synthase (NOS) isoforms including (nNOS, eNOS and iNOS) are expressed in brain throughout ageing and associated pathologies (18-21). iNOS is localized in the dentate gyrus and CA1 region of hippocampus that were identified by immunohistochemistry (IHC) studies against iNOS (22). Numerous behavioral and molecular studies indicate that one of the primary causes of cognitive impairments is cholinergic dysfunction (8, 22, 23). Also it has been reported that the increase of iNOS expression during hypoxia impairs the memory formation by affecting the cholinergic functions via alteration of acetyl cholinesterase activity (8). Moreover, it has been demonstrated that iNOS inhibitors such as aminoguanidine (AG) can ameliorate cholinergic system dysfunctions induced by amyloid beta (A*β*) injections (22).

The N-methyl-D-Aspartate (NMDA) receptor plays an important role in synaptic plasticity and behavioral learning and memory (24–26), because of its high concentration in the hippocampus, cortex and striatum, the brain regions that were necessary for spatial learning and memory (27, 28). 

The aim of the present work was to study the effects of intra-hippocampal infusion of 1400W as a selective iNOS inhibitor in cannulated non-anesthetized and non-cannulated anesthetized animals on spatial memory in Morris water maze.

## Experimental

*Animals*

Male Albino Wistar rats (180-220 g) were obtained from faculty of pharmacy of Tehran University of Medical Sciences, housed in groups of five in each stainless-steel cages, and given food and water *ad libitum *under a standard 12 h light/12 h dark cycle. The animals were trained and tested during the light cycle. All procedures were carried out in consistent with the guidelines for the Care and Use of Laboratory Animals, Tehran University of Medical Sciences. All efforts were made to create light of suffering and to trim down the number of animals used in this study.

*Drugs*

1400W (CALBIOCHEM^®^, Merck KGaA, Darmstadt, Germany) was dissolved in deionized water. Ketamine (alfasan, Holland) and xylazine (Pantex Holland B.V.) were used for surgical anesthesia. Other chemicals and materials were obtained from commercial sources.

*Behavioral training and testing*

In this study, 4-day training trials of animals in the Morris water maze task were performed. 1400W was administered immediately after last trial of training in fourth day and spatial memory was tested 48 h after the infusions of 1400W. Spatial memory retention was tested in this task by measuring escape latency, traveled distance, and swimming speed parameters with EthoVision system which was bought from Noldus Information Technology company (Wageningen, the Netherlands), as described in our previous studies (4-7). The testing step included 1 block of 4 trials. 

*1400W microinjections*

The animals were anesthetized with Ketamine (80 mg/kg) and Xylazine (20 mg/kg) to get ready for stereotaxic surgeries. In cannulated rats, one week after recovery from surgery, the training of the animals was started in Morris water maze task. 1400W (10, 50 and 100 μM/side), was microinjected bilaterally in a volume of 1 μL/side into the CA1 region of hippocampus via cannulas placed 3.8 mm posterior, 2.2 mm lateral to bregma and 2.7 mm ventral to the surface of the skull consistent with the atlas of Paxinos and Watson (29). ‘In non-cannulated rats, bilateral infusions were performed directly via a Hamilton syringe (1 μL/side) into the CA1 region of the hippocampus in anesthetized rats.’ In all groups, 1400W was infused immediately after last trial of training in fourth day. The control groups received deionized water. 

*Statistics*

One-way analysis of variance (ANOVA) was used for comparison of behavioral data. A Newman–Keuls multiple comparison post hoc test was employed to assess differences in behavioral scores. T-test was also used to compare the statistical differences between cannulated and non-cannulated groups. A p-value of 0.05 or less was considered statistically significant.

## Results

*Effects of four days training in cannulated animals after surgical recovery and intact (non-cannulated) rats before anesthesia*

In this study, all animals including control groups and animals selected to receive bilateral infusions of 1400W in anesthetized (non-cannulated) and non-anesthetized (cannulated) conditions, trained completely after four days of training in the Morris water maze task as pointed out by reduction in time and distance for finding the hidden platform (Table 1). There were significant differences between the first and fourth days of training in finding the hidden platform in terms of escape latency and traveled distance in cannulated (**p < 0.01) and non-cannulated (***p < 0.001) animals. Also, there were not any significant differences between the cannulated and non-cannulated animals in spatial learning parameters during training period. Significant difference was not observed in swimming speed due to the training trials in any of the animal groups (Table 1).

**Table. 1:** Effects of four days training on escape latency, traveled distance and swimming speed in cannulated and non-cannulated animals

**Swimming speed (cm/sec)**		**Traveled distance (cm)**		**Escape latency (sec)**		**Training days**
Non-cannulated	cannulated	Non-cannulated	cannulated	Non-cannulated	cannulated	
18.3±1.8	17.93±1.5	862.8±129	658.4±21.1	42.8±5.9	29.8±4.2	**Day 1**
21.2±1.7	24.4±0.7	210.5±59***	200.3±49**	11.63±2.5***	8.6±2.2**	**Day 4**

Four days trained animals learned well to find the hidden platform in MWM. The table shows that both cannulated (non-anesthetized) and non-cannulated (anesthetized) animals learned how to find the hidden platform. There were significant differences (**p < 0.01 and ***p < 0.001, respectively) between the first and fourth days of training in MWM in escape latency and traveled distance parameters for cannulated and non-cannulated rats. There was not a significant difference for speed of swimming between the first and fourth days of training in all animals. The results are presented as Mean ± SEM for 8 animals in each group.

*Effects of 1400W microinjection on time and distance of finding the hidden platform during testing trials in cannulated and non-cannulated rats*

**Figure 1:**
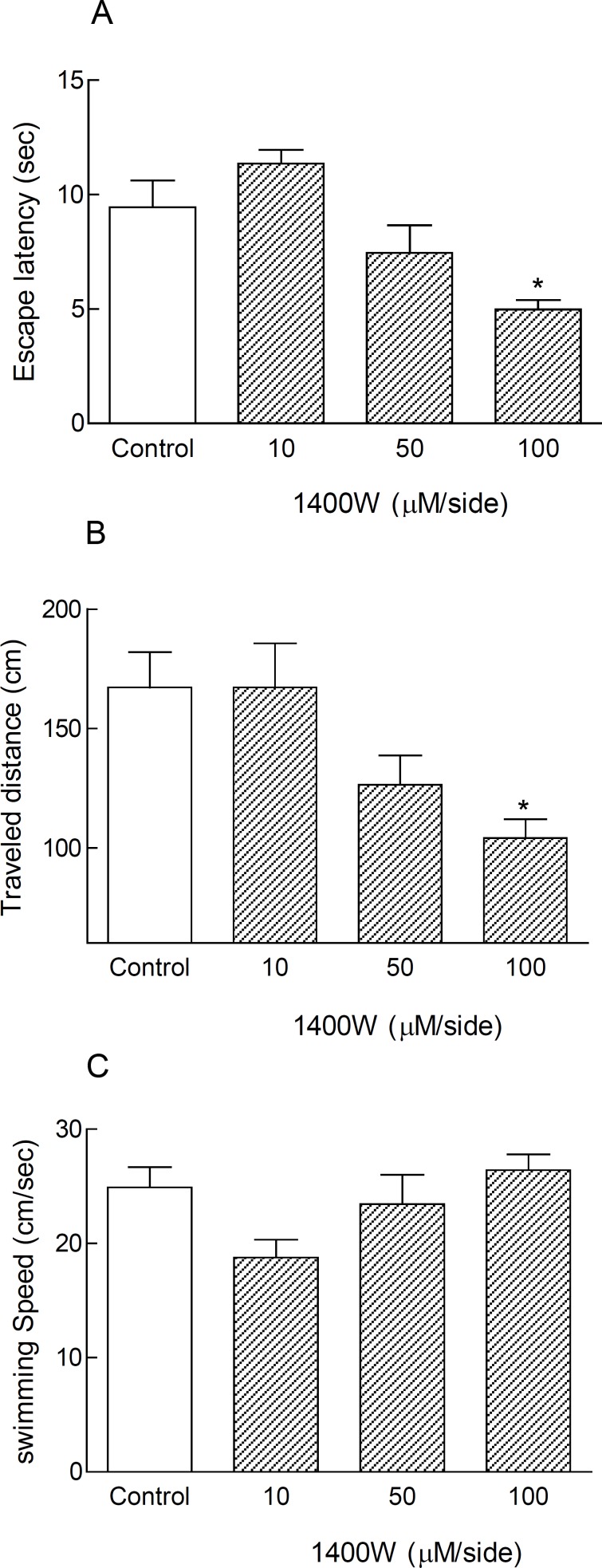
Treatment with 1400W as a selective iNOS inhibitor caused spatial memory improvement in cannulated non-anesthetized animals in MWM during testing trials. Inhibition of inducible nitric oxide synthase by bilateral intra-hippocampal infusion of 1400W (100 μM/side) via cannulas after surgical recovery, led to significant decrease in escape latency and traveled distance (*p < 0.05) in comparison with control group (Figures 1A and B). The swimming speed did not change significantly in all treated animals (Figures 1C). Each bar graph shows Mean ± SEM for 8 animals in each group

Post-training bilateral microinjections of 1400W (10, 50 and 100 μM/side) into the CA1 region of the hippocampus directly via a Hamilton syringe did not change the time, distance, and speed of finding the hidden platform in anesthetized animals (non-cannulated rats) in comparison with control group (Table 2). But, bilateral intra-hippocampal infusions of this agent via a cannula with dose of 100 μM/side after surgical recovery in consciousness condition led to significant reduction in escape latency and traveled distance (*p < 0.05) (Figures 1A and B). The swimming speed was similar in all groups, representing no motor disturbances in all treated animals (Table 2 and Figure 1C).

**Table 2:** Effects of 1400W infusions on spatial memory in non-cannulated (anesthetized) animals in Morris water maze

**Treatment (μM/side)**	**Escape latency (sec)**	**Traveled Distance (cm)**	**Swimming Speed (cm/sec)**
**Deionized Water**	14.8 ± 2.6	341.2 ± 53.05	24.9 ± 1.2
**1400W 10 μM**	12.5 ± 1.8	313.1 ± 43.6	23.8 ± 1.3
**1400W 50μM**	9.3 ± 0.99	193.6 ± 23.7	21.3 ± 1.43
**1400W 100 μM**	9.9 ± 0.54	207.6 ± 21.1	21.2 ± 1.5

Bilateral direct infusion of 1400W in CA1 region of hippocampus in anesthetized rats did not alter spatial memory significantly during testing trials. Post-training bilateral infusions of 1400W (10, 50 and 100 μM/side) into the CA1 region of the hippocampus directly via a Hamilton syringe in stereotaxic surgery instrument did not alter the time, distance, and swimming speed of finding the hidden platform considerably in anesthetized animals in comparison with control group (Table 2). The results are presented as Mean ± SEM for 8 animals in each group

**Figure 2:**
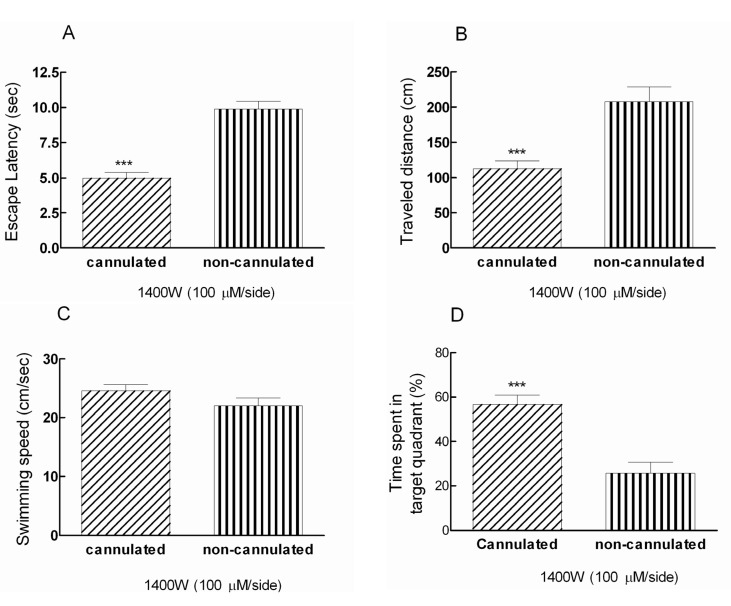
Post-training bilateral intra-hippocampal infusions of 1400W (100 μM/side) decreased the escape latency and traveled distance significantly (***p < 0.001) in cannulated animals compared to non-cannulated (anesthetized) group (Figure 2A and 2B). There is a significant difference (***p < 0.001) between cannulated and non-cannulated rats in time spent in target quadrant (Figure 2D).

Comparison effects of Post-training administration of 1400W with dose of 100 μM/side between animals in anesthesia condition and post-recovery phase showed significant decrease in escape latency and traveled distance (***p < 0.001) in consciousness rats (Figures 2A and B). Moreover, the swimming speed was the same in treated animals (Figure 2C).

In addition, the presence time of recovered animals during testing trials in target quadrant; the quadrant contains the hidden platform, was 56.7% in comparison with animals that received 1400W during anesthesia (25.8%) (Figure 2D). In fact, there is a significant difference (***p < 0.001) between cannulated and non-cannulated rats in time spent in target quadrant (Figure 2D) 

## Discussion

In our work, we evaluated spatial memory by using Morris water maze, because performing the spatial learning during training and testing trials in this task requires the hippocampal neural pathways (4–7, 30). In this study, we found no significant differences in escape latency, traveled distance and swimming speed between cannulated animals and intact rats during four days training period. This result confirmed that in both groups of animals, training was completed properly. 

In addition post training bilateral infusions of iNOS inhibitor via cannula in recovered animals or by Hamilton syringe in anesthetized rats did not reveal any significant differences in swimming speed compared with control groups. These observations indicating that 1400W did not induce any motor dysfunctions. Such results give credence to our finding that spatial memory retention improvement is caused by iNOS inhibitor. 

One of the important findings of the present investigation is that high dose infusion of 1400W caused a considerable enhancement on spatial memory in recovered cannulated animals compared with administration of 1400W in anesthetized rats. There is evidence that shows the involvement of different isoforms of NOS in memory function. The use of specific nNOS inhibitor induced deficits in early olfactory associative learning in MWM and radial maze (31, 32). Previous published reports indicated that inhibition of eNOS caused memory impairment in chicks (33, 34). In contrast, the role of iNOS inhibitor in attenuation of A*β*-induced memory impairment has also been shown by some investigators (22). They found that infusion of A*β*1-40 in the brain, induced iNOS expression which is accompanied with memory loss (22). Also it has been demonstrated that increase in A*β*-induced iNOS expression cause cholinergic system dysfunction (22). The interactions between iNOS and AChE activity was also reported in other studies (8). In the present experiment because of a more invasive drug administration in non-cannulated (anesthetzed) animals compared to the classically cannulated rats, it is possible that memory improvement induced by 1400W was caused partially by interaction with cholinergic function.

As stated earlier, inducible NOS is a calcium-independent which mediate immune function of NO (16). In addition, the effects of anesthesia and mechanical trauma produced by the foreign object like a needle induce an acute inflammatory immune response that increases the expression of iNOS (35). Thus, it is reasonable to assume that non significant improvement of spatial memory we observed in testing trials of non-cannulated animals infused with 1400W during anesthesia was caused by an increase in iNOS levels. 

Nitric oxide as a component of the various neurotransmitter pathways is involved in neural plasticity contributing to memory in different areas of brain including the hippocampus (16). The NO/cGMP pathway is influenced by anesthesia (36). Among the anesthetics affecting the NO pathway, ketamine that used in combination with xylazine as an analgesic is widely reported in the published documents and literatures (36). Ketamine-induced cGMP accumulation has been observed in the CNS that suggested its action on the neuronal nitric oxide pathway (36-38). Ketamine is a non-competitive blocker of the glutamate subtype of the *N*-methyl-D-Aspartate (NMDA) receptors (24, 36). NMDA receptors that play an important role in neural physiology, synaptic plasticity and behavioral learning and memory (24, 36) are concentrated in the hippocampus (24, 27, 28, 37). A considerable body of evidence also shows the impairment effects of NMDA-receptor blockers such as ketamine in different kinds of memory (24). Since in our study, we tested the animals for evaluation of spatial memory retention 48 h after 1400W infusions, therefore it is reasonable to deduce that in anesthetic rats the impairment effects of ketamine still remained during testing trials. Also it is possible in anesthetized animals ketmine-induced hippocampal iNOS increase after 48 h was not inhibited by 100 μM/side of 1400W sufficiently. Moreover, although the effects of ketamine-induced nitric oxide in the brain are somewhat conflicting, caution should be noted when dealing with learning and memory function in which NO may play an important role. In addition, our findings suggest that ketamine can affect receptors, membranes, ion channels, neurotransmitters, brain blood flow and metabolism in memory processes. Also based on the time of spatial memory evaluation after anesthesia, several important factors such as type and distribution of neurotransmitters, metabolic function, capacity for plasticity, depth of anesthesia and root of administration may show different susceptibility to ketamine-mediated changes. The involvement of cAMP/PKA signaling in relationship between anesthesia and memory in Drosophila has been reported by Tanaka *et al*. (14). They demonstrated that many mutants of general anesthesia and those of memory are overlapped suggesting that common molecules and signal pathways are involved in both phenomena (14). We previously showed that cAMP/PKA signaling has important function in spatial memory (5-7). In addition, behavioral studies in Aplysia California, confirmed the pivotal function of cAMP/PKA signaling in the short and long-lasting forms of learning and memory (7, 39-41). Therefore, it is possible that ketamine via affecting on PKA and inhibition of cAMP/PKA pathway prevented the 1400W-induced memory improvement in anesthetic rats. It is also proposed that cAMP/PKA pathway would increase cholinergic activity (5, 7, 42–44). Thus, it is probable that ketamine via affecting cAMP/PKA signaling decrease cholinergic function and attenuated memory improvement of 1400W in anesthetic animals. 

In conclusion, our findings and those of others provide documents in support of the interacting effects of anesthesia and iNOS inhibitors on the learning and memory processes in animals.

Finding the exact cellular, molecular and neurotransmitters mechanism (s) of these results requires more knowledge of anesthetic agents, 1400W and cAMP/PKA pathway roles in learning and memory, which should be obtained in our future experiments.
